# Pathophysiology of Inflammatory Bowel Disease: Innate Immune System

**DOI:** 10.3390/ijms24021526

**Published:** 2023-01-12

**Authors:** Angela Saez, Beatriz Herrero-Fernandez, Raquel Gomez-Bris, Hector Sánchez-Martinez, Jose M. Gonzalez-Granado

**Affiliations:** 1 LamImSys Lab, Instituto de Investigación Sanitaria Hospital 12 de Octubre (imas12), 28041 Madrid, Spain; 2 Facultad de Ciencias Experimentales, Universidad Francisco de Vitoria (UFV), 28223 Pozuelo de Alarcón, Spain; 3 Departamento de Fisiología, Facultad de Medicina, Universidad Autónoma de Madrid (UAM), 28029 Madrid, Spain; 4 Department of Immunology, Ophthalmology and ENT, School of Medicine, Universidad Complutense de Madrid (UCM), 28040 Madrid, Spain; 5 CIBER de Enfermedades Cardiovasculares (CIBERCV), 28029 Madrid, Spain; 6 Centro Nacional de Investigaciones Cardiovasculares (CNIC), 28029 Madrid, Spain

**Keywords:** innate immune system, inflammatory bowel disease, ulcerative colitis, Crohn’s disease, neutrophil, macrophage, dendritic cell, intestinal homeostasis

## Abstract

Inflammatory bowel disease (IBD), comprising Crohn’s disease (CD) and ulcerative colitis (UC), is a heterogeneous state of chronic intestinal inflammation with no exact known cause. Intestinal innate immunity is enacted by neutrophils, monocytes, macrophages, and dendritic cells (DCs), and innate lymphoid cells and NK cells, characterized by their capacity to produce a rapid and nonspecific reaction as a first-line response. Innate immune cells (IIC) defend against pathogens and excessive entry of intestinal microorganisms, while preserving immune tolerance to resident intestinal microbiota. Changes to this equilibrium are linked to intestinal inflammation in the gut and IBD. IICs mediate host defense responses, inflammation, and tissue healing by producing cytokines and chemokines, activating the complement cascade and phagocytosis, or presenting antigens to activate the adaptive immune response. IICs exert important functions that promote or ameliorate the cellular and molecular mechanisms that underlie and sustain IBD. A comprehensive understanding of the mechanisms underlying these clinical manifestations will be important for developing therapies targeting the innate immune system in IBD patients. This review examines the complex roles of and interactions among IICs, and their interactions with other immune and non-immune cells in homeostasis and pathological conditions.

## 1. Introduction

Inflammatory bowel disease (IBD) encompasses a group of heterogeneous diseases that entail chronic, relapsing gastrointestinal tract inflammation of inexactly known etiology and pathogenesis. IBD etiology may involve the host immune system, genetic variability, and environmental factors [1]. IBD is clinically classified as Crohn’s disease (CD) or ulcerative colitis (UC) based on symptoms, disease location, and histopathological characteristics. UC causes long-lasting inflammation and superficial ulcerative disease in the colon, whereas CD is a transmural disease often associated with granuloma formation and can appear in any part of the gastrointestinal tract [2,3,4,5]. IBD can be associated with life-threatening conditions, including primary sclerosing cholangitis, blood clots, and colon cancer [6]. IBD is usually diagnosed between the ages of 20 and 40 years, but can start at any age. IBD shows alternating periods of clinical relapse and remission.

The intestinal mucosa is composed of epithelial cells, goblet and Paneth cells, stroma and immune cells. The intestinal epithelium includes a monolayer of epithelial cells closely bound by tight junctions and interposed with immune cells. The intestine is structured as a series of protrusions known as villi and invaginations called crypts of Lieberkühn [7]. The epithelium participates in nutrient absorption and, at the same time, interposes a physical barrier to the contents of the intestinal lumen. The epithelium also interacts with the intestinal microbiota and the immune system, sending receiving signals to and from both.

The epithelium includes goblet and Paneth cells, which, respectively, produce mucus and antimicrobial peptides, thus limiting the spread of luminal microorganisms [7]. A marked reduction in goblet cell numbers has been linked to a loss of mucus layer thickness in Crohn’s disease [8], and abnormal mucus composition has been reported in UC [9]. Beneath the epithelium, the lamina propria contains stromal cells, including fibroblasts, myofibroblasts, and perivascular pericytes. These cells serve functions in fibrosis and wound healing [7], and may be related to the aggravation of UC through their capacity to produce chemokines, including chemokine (C-C motif) ligand (CCL)19, CCL21, and the immune-system regulator interleukin (IL)-33 [10].

Plasma cells release immunoglobulin (Ig)A, inhibiting the infiltration of pathogenic microorganisms and helping to sustain a homeostatic equilibrium between the host and commensal microbiota. Both the epithelium and other non-immune intestinal components are important mediators of intestinal homeostasis and IBD pathophysiology, reviewed in [11,12]. However, some of the functions of these non-immune cells are mediated through interaction with components of the immune system, as will be described in this review.

The immune system confers host defense against pathogens and provides anti-tumor protection. At the same time, regulatory mechanisms counterbalance these responses to prevent reactions against self and innocuous external antigens, thus promoting a state of tolerance.

The immune system can be classified into innate and adaptive immunity. Innate immunity, composed of myeloid cells among other elements, initiates rapid and nonspecific responses to conserved structural motifs on microorganisms. Innate immune cells (IIC) express pattern recognition receptors (PRRs), such as toll-like receptors (TLRs) and Nod-like receptors (NLR), allowing them to distinguish pathogen-associated molecular patterns (PAMPs) and damage-associated molecular patterns (DAMPs). IIC promote host defense and inflammation by generating cytokines and chemokines, triggering the complement cascade and phagocytosis, or stimulating adaptive immunity by presenting antigens. Notable IIC include neutrophils, monocytes, macrophages, and dendritic cells (DCs) [13,14].

## 2. IBD Pathophysiology

The etiology of IBD remains elusive, but IBD appears to be sustained in genetically susceptible individuals by an impaired immune response against intestinal microorganisms. This abnormal immune response is associated with dysregulation of both innate and adaptive immune responses.

IBD features breach the epithelial barrier of specific zones in the intestine, and non-resolving mucosal damage is thought to be an important characteristic of the disease [15]. While generally unknown, the cause of this damage could be related to an infectious agent [16], a chemical compound [1], or a metabolic alteration probably related to diet-mediated dysbiosis [17]. The disease is then thought to be perpetuated by deficient resolution of the inflammatory response to this initial injury [18]. Unsuccessful resolution of inflammation is possibly supported by disruption of tolerance towards commensal microorganisms or autologous signals of tissue damage [15,19].

There is also some uncertainty as to whether the epithelial barrier alterations precede or follow the development of inflammation in the lamina propria [20].

### 2.1. Innate Immune Cells in the Pathogenesis of IBD

In IBD, the innate immune system is the first responder to PAMPs and to molecules released from damaged or dying cells, known as DAMPs. DAMPs and PAMPs activate the innate immune system by interacting with PRRs. These patterns can be sensed by several components of the innate immune system, including granulocytes, neutrophils, monocytes, myeloid-derived suppressor cells, macrophages, and dendritic cells. In addition, these patterns can also be recognized by non-immune cells, such as intestinal epithelial cells (IECs) and myofibroblasts.

IICs respond to these signals, temporarily enhance the epithelial barrier, and clean up the effects of inflammation [21]. The implication of IIC in IBD is the focus of this review.

#### 2.1.1. Neutrophils in Gut Homeostasis

Neutrophils are the most numerous immune cells in the human circulation and are quickly recruited to sites of infection or inflammation [22], forming the first line of immune defense.

**Figure 1:**
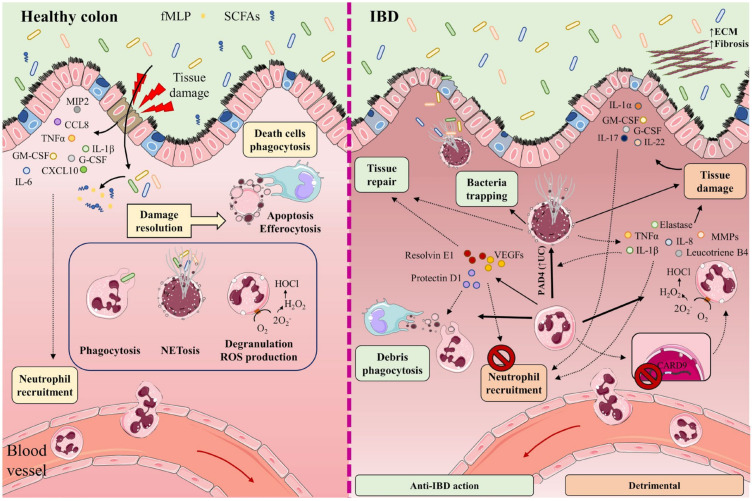
In healthy intestine (**left**), damage to the intestinal barrier triggers the recruitment of neutrophils from the circulation to the inflamed tissue along a chemotactic gradient formed by cytokines (IL-1β, IL-6, TNF-α), chemokines (CCL8, CXCL10, MIP-2), and growth factors (GM-CSF, G-CSF). Neutrophil recruitment is also mediated by bacteria-derived molecules such as formyl-methionyl-leucyl-phenylalanine (fMLP) and short-chain fatty acids (SCFAs). The recruited neutrophils participate in the elimination of microorganisms through phagocytosis, degranulation, reactive oxygen species (ROS) generation, and the release of neutrophil extracellular traps (NETs). Once their functions are completed, neutrophils undergo apoptosis and efferocytosis, facilitating the resolution of inflammation, tissue repair, and a return to normal tissue homeostasis. The participation of neutrophils and NETs in IBD is a double-edged sword (**right**). Neutrophils cooperate in wound healing and the resolution of inflammation by releasing vascular endothelial growth factors (VEGFs) and lipid mediators (protectin D1, resolvin E1). These factors impede neutrophil recruitment and promote phagocytosis. NETs impede the spread of microorganisms by trapping them in an environment of microbicidal components and stimulate the healing of the intestinal mucosa. Neutrophils directly cause tissue damage by releasing neutrophil elastase, proteases (MMPs), pro-inflammatory cytokines (IL-8, TNF-α, IL-1β), leukotriene B4, and ROS. These factors provoke not only injury to the epithelial barrier, but also the recruitment of neutrophils and other immune cells to the inflamed tissue. Neutrophil recruitment is also promoted by the cytokines IL-1α, IL-17, IL-22, G-CSF, and GM-CSF. Lack of the IBD protective gene CARD9 in neutrophils enhances ROS generation. IL-8, TNF-α, and PAD4 (increased in UC patients) contribute to NET production. Accumulation of NETs in the colon is accompanied by the induction of tissue damage and inflammation, as NETs also boost TNF-α and IL-1β production. Part of the figure was generated by using pictures from Servier Medical Art.

When the intestinal barrier is damaged, neutrophils are recruited from the circulation to the inflamed tissue through a plethora chemotactic gradients formed by cytokines such as IL-1β, IL-6, and tumor necrosis factor (TNF)-α; chemokines such as CCL8, chemokine (C-X-C motif) ligand (CXCL)10, and macrophage inflammatory protein 2 (MIP)-2 (also known as CXCL2); and growth factors such as granulocyte-macrophage colony-stimulating factor (GM-CSF) and granulocyte colony-stimulating factor (G-CSF) [23,24,25]. Neutrophil recruitment is also mediated by bacteria-derived molecules such as formyl-methionyl-leucyl-phenylalanine and short-chain fatty acids (SCFAs) [26,27,28] (Figure 1).

Neutrophils participate in the elimination of microorganisms through phagocytosis, degranulation, the generation of reactive oxygen species (ROS), and the release of neutrophil extracellular traps (NETs). NETs are mesh-like structures made of DNA and its histone scaffold together with granule components such as myeloperoxidase (MPO), cathepsin G, neutrophil elastase, and protease 3. NETs protrude from the membrane of the activated neutrophil to restrain large microorganisms, activate complement factors, and therefore facilitate contained lysis through their bactericidal and permeability-increasing actions [29,30,31,32]. Once their functions are completed, neutrophils undergo apoptosis and efferocytosis, facilitating resolution of the inflammatory response, repair, and a return to normal tissue homeostasis [33,34,35].

#### 2.1.2. Neutrophils in the Gut during IBD

Some studies have described the participation of NETs in IBD as a double-edged sword. On the one hand, NETs can impede the spread of microorganisms by trapping them in an environment of microbicidal components, while also stimulating the healing of the intestinal mucosa upon injury and helping to sustain the stability of the intestinal epithelium [36,37]. On the other hand, increased neutrophil activity and exacerbated NETs production can impair intestinal mucosal barrier function, damage the intestinal epithelium, and accentuate disease symptoms [38]. Neutrophils directly promote tissue damage by releasing proteases such as matrix metalloproteinases (MMPs) and neutrophil elastase, and by altering membrane properties by releasing ROS [39] (Figure 1).

Proteomics studies and microscopy validations have identified eleven neutrophil- and NETs-associated proteins with increased abundance in biopsies from UC patients [40], and similar results have been reported by others [39].

Dextran sodium sulfate (DSS)-induced colitis in mice promotes the accumulation of NETs in the colon, accompanied by the induction of epithelial cell death by apoptosis, breakage of tight junctions, increased permeability, and augmented bacterial translocation and inflammation [41]. Moreover, NETs accumulation boosts TNF-α and IL-1β production in plasma by signaling via the ERK1/2 pathway. The reduction of NETs protects against colitis and inhibits the augmentation of pro-inflammatory factors implicated in IBD [41].

An increase in neutrophil activity has been observed in IBD patients [42]. This increase is associated with the release of TNF-α and the presence of lipopolysaccharides, two factors that contribute to neutrophil activation. Other factors that can contribute to NETs production are IL-8 produced by endothelial cells, and an increased abundance of protein arginine deiminase 4 (PAD4) [30,31,43]. PAD4 is more abundant in intestinal tissue from UC patients than healthy individuals [31], and in damaged tissue rather than healthy tissue from the same individual [30,31,32]. PAD4 mediates histone citrullination, an important event in NETosis that precedes chromatin decondensation and DNA release.

An analysis of NETs-associated proteins in colon samples from patients with UC, CD, and colon cancer showed a greater abundance of PAD4, MPO, neutrophil elastase, and citrullination histone H3 (CitH3) in UC than in CD. Moreover, neutrophils in UC tissues produced more NETs upon treatment with TNF-α [44]. In a similar way, increased expression of Ly6G, CitH3, and PAD4 has been found in mouse colon in a model of colitis induced by 2,4,6-trinitrobenzene sulfonic acid (TNBS) [45]. This effect was associated with damage to the intestinal epithelial barrier [45]. Reduced NETs formation ameliorated colitis symptoms and tissue damage [46,47,48,49].

Neutrophils promote IBD gut inflammation by producing high levels of ROS that impair the epithelial barrier and promote redox-sensitive inflammatory pathways [50]. The epithelial barrier is also damaged by neutrophil-produced proteases, pro-inflammatory cytokines such as IL-8, TNF-α, and leukotriene B4, which additionally recruit monocytes and more neutrophils to the inflamed tissue [51,52].

Another cytokine recently proposed to promote neutrophil recruitment to colonic tissue is IL-22, whose levels correlate with neutrophil infiltration [53]. Neutrophils can be activated by the cytokines IL-1β and IL-18 [54], produced during inflammasome assembly [55,56], and upon the release by necrotic cells of the nuclear ‘alarmin’ IL-1α [57]. Other mediators of neutrophil action altered in IBD include GM-CSF and G-CSF [58] and IL-17A and IL-17F [59], which act through the IL-23–IL-17A–G-CSF axis [60,61], providing a possible explanation for the continuous regeneration of neutrophils in IBD [15]. Neutrophils also express the IBD-protective gene caspase recruitment domain 9 (Card9), which provides them with the capacity to protect against DSS-induced colitis; a lack of CARD9 enhances mitochondrial dysfunction and ROS generation, leading to neutrophil apoptosis and increased inflammation [62].

The intestinal epithelium constitutes a physical barrier that isolates subepithelial tissues from luminal contents, providing a fundamental support for intestinal homeostasis. Neutrophils are associated with bystander tissue damage, but also play a role in epithelial restitution [63].

As well as killing microorganisms, neutrophils cooperate in wound healing and the resolution of inflammation by releasing vascular endothelial growth factors and beneficial lipid mediators such as protectin D1 and resolvin E1. These factors impede further neutrophil recruitment and augment the phagocytosis of apoptotic neutrophils by macrophages. Neutrophils also remove cellular debris from sites of inflammation by phagocytosis [61,64,65,66]. The release of proteases within NETs can regulate cytokine function by proteolysis [67], and this phenomenon might also regulate cytokines in IBD.

Contrasting these protective effects, the accumulation of hyperactivated neutrophils promotes an alteration of crypt structure and the formation crypt abscesses. This process features a disproportionate enzymatic reaction, generation of the pro-inflammatory cytokines TNF-α and IL-1β, and the secretion of the non-cytokine inflammatory molecules α defensins and calprotectin, attracting monocytes, T cells, and more neutrophils to the inflammation site and promoting the pathogenesis of IBD [68,69,70,71,72]. Neutrophils can also promote goblet-cell depletion, a main feature of IBD [73,74,75].

Neutrophils are also, themselves, regulated by the effects of intestinal epithelial cells during intestinal inflammation, a topic recently reviewed in [63].

In summary, neutrophils play intricate roles in intestinal inflammation, contributing to the elimination of invading pathogens and epithelial restitution, while at the same time participating in the disruption of crypt architecture and generating bystander tissue damage, roles that impede and promote the development of the IBD, respectively.

Under the influence of neutrophils, other phagocytic cells such as monocytes and macrophages remove cell debris and help to distinguish damaged areas from tissue areas less affected by acute inflammation [76].

#### 2.1.3. Macrophages in the Gut in Steady State Conditions

**Figure 2:**
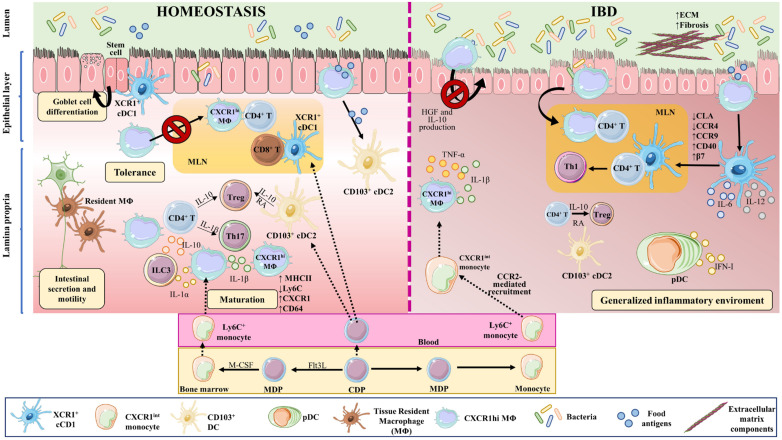
Macrophages and dendritic cell in homeostasis and IBD. Macrophages and DCs play important roles in homeostasis and in the development of IBD by phagocytosing cellular debris, producing cytokines, regulating tissue repair, and interacting with other cells. In the intestine, monocyte-derived macrophages (MΦ) are more abundant than tissue-resident macrophages of embryonic origin. Both perform phagocytosis, produce cytokines, and interact with other cells. Upon weaning, bone marrow-derived monocytes egress from the circulation and extravasate into the tissue, where they undergo differentiation and maturation (downregulation of Ly6C, production of MHCII, and increased expression of CX3CR1). In homeostasis, tissue-resident macrophages in the muscularis externa interact with enteric and myenteric neurons controlling intestinal secretion and motility, while in the lamina propria, macrophages provide signals to intestinal stem cells that give rise to goblet cells, Paneth cells, and intestinal epithelial cells. These macrophages also modulate T cell activities and functions, via the secretion of IL-10 for Tregs and IL-1β for Th17 cells. In addition, they affect ILC3 cells through the production of IL-1α and IL-1β. The migration of antigen-loaded CX3CR1^high^ intestinal macrophages to mesenteric lymph nodes is impaired by intestinal microbiota, thus affecting antigen presentation to T cells and effectively sustaining tolerance towards commensal bacteria. On the other hand, XCR1^+^ DCs play a tolerogenic role upon recognizing commensal bacterial components, while CD103^+^ cDC2s seem to be important for initiating oral tolerance through their capacity to generate RA and IL-10. In IBD, large numbers of Ly6C^high^ inflammatory monocytes are recruited to the intestine in a CCR2-dependent manner, becoming pro-inflammatory effector cells. These inflammatory macrophages produce TNFα, IL-6, and iNOS, and directly cause the onset and development of fibrosis through a disproportionate accumulation of ECM. The intestinal microbiota is impaired during chronic colitis, and CX3CR1^high^ macrophages can change their habits and migrate to lymph nodes. CD103^+^ cDC numbers are significantly reduced in the inflamed and uninflamed intestine in IBD; however, activated DCs can release inflammatory cytokines, in addition to type I IFN produced by pDCs. All these phenomena contribute to a generalized inflammation. Part of the figure was generated by using pictures from Servier Medical Art.

Macrophages are highly plastic cells, and their functions depend on their developmental ontogeny and surrounding environment [77,78,79] (Figure 2). During embryonic development, self-maintaining tissue-resident macrophages derive from the yolk sac and fetal liver progenitors [80,81,82,83]. After birth, blood circulating bone marrow-derived monocytes are recruited to tissues, where they differentiate to macrophages, replenishing tissue-resident populations and adopting a phenotype conditioned by the local tissue environment [82,84,85,86].

Both tissue-resident and monocyte-derived macrophages perform the typical macrophage functions of phagocytosis, cytokine production, and host interaction [87]. In the intestine, monocyte-derived macrophages are more abundant than macrophages of embryonic origin [77]; however, distinct subsets of embryonic-derived macrophages remain in the intestine even after weaning [88].

Upon weaning, bone marrow-derived monocytes egress from the circulation and extravasate into the tissue, contributing to macrophage replenishment in the intestine [89]. After extravasation, monocytes undergo differentiation and maturation processes, acquiring a more macrophage-like phenotype. These changes include downregulation of Ly6C generation, production of class II major histocompatibility complex molecules (MHCII), and increased expression of C-X3-C motif chemokine receptor1 (CX3CR1); monocyte-to-macrophage transition thus involves a switch from a Ly6C^high^, CX3CR1^int^ state to a mature Ly6C^−^, CX3CR1^high^, CD64^+^, MHCII^+^ state [77,90,91].

In steady-state conditions, intestinal macrophages are distributed throughout the gut structure, including the lamina propria, submucosa and the muscularis externa [92].

Distal to the gut lumen, in the muscularis externa, are located long-lived, bipolar, and stellate self-renewing embryonic-derived macrophages [93]. Muscularis macrophages interact with enteric and myenteric neurons, influencing enteric neurons [94], and controlling intestinal functions including secretion and motility [95,96,97].

The macrophages of the lamina propria are rounded monocyte-derived cells that are constantly replenished [89,94]. These macrophages are short lived and are distributed close to the lumen, where they constantly encounter the gut microbiota [93,98] and contribute to oral tolerance [99,100]. In this layer, macrophages provide signals to the intestinal stem cells, which give rise to goblet cells, Paneth cells, and intestinal epithelial cells [101,102]. These macrophages also modulate regulatory T cell (Treg) activity and function via the secretion of IL-10 [103] and T helper (Th)17 cells by providing IL-1β [104]. Lamina propria macrophages thus support intestinal homeostasis through a mix of phagocytic and antibacterial functions, immune modulation, and tissue repair [105,106].

Intestinal-tissue macrophage phenotypes and functions depend on the microbiota and their metabolites and on microenvironmental cues [77,107,108,109]. Intestinal macrophages can thus be classified into three distinct populations according to their homeostatic function: host defense, wound healing, and immune regulation [110].

Host defense macrophages have microbicidal activity and, upon stimulation with interferon (IFN)γ or TNF-α, generate cytokines that are commonly secreted by T cells, natural killer cells, or antigen presenting cells (APC). Wound healing macrophages develop upon contact with IL-4 released by T cells or granulocytes, and play a role in tissue repair. Regulatory macrophages, which have an anti-inflammatory function, are generated in response to several stimuli, including IL-10, glucocorticoids, and apoptotic cells [110].

Intestinal macrophages need to combat invading pathogenic bacteria while tolerating beneficial probiotic bacteria [111]. To discern between commensal and harmful bacteria, macrophages recognize harmful bacteria through PRRs such as TLRs and NOD-like receptors (NLRs) [110,112,113]. Intestinal macrophages are excellent phagocytes of harmful bacteria, but upon engulfing or recognizing harmful bacteria they produce low amounts of pro-inflammatory cytokines [114,115,116,117]. In contrast, they naturally produce elevated amounts of IL-10, which is associated with their reduced response to TLR-triggering [103,114,118,119,120,121,122], suggesting a somewhat anti-inflammatory phenotype for resident intestinal macrophages, which avoid bacteria-activated inflammation in the gut under steady state conditions [117,123].

In the gut, IL-34 and CSF-1 (also called M-CSF)—both ligands for the CSF-1 receptor (CSF-1R)—promote monocyte and macrophage differentiation [124,125], and mice lacking CSF-1 or CSF-1R are deficient for tissue macrophages [126,127,128,129]. Supporting this, administration of anti-CSFR antibodies reduces macrophage numbers [130], while recombinant CSF-1 increases intestinal macrophage infiltration [131].

Intestinal macrophages are involved in phagocytosis and the clearance of apoptotic cells [132,133], including apoptotic IECs, helping to maintain epithelial barrier integrity under steady state conditions [134].

CX3CR1^high^ intestinal macrophages sense and take up bacterial antigens from the intestinal lumen through their transepithelial dendrites [135,136,137,138,139,140]. In homeostasis, intestinal microbiota inhibit the migration of antigen-loaded CX3CR1^high^ intestinal macrophages to mesenteric lymph nodes, thereby also inhibiting antigen presentation to T cells and effectively sustaining tolerance towards commensal bacteria. When the intestinal microbiota is disturbed or under chronic colitis conditions, CX3CR1^high^ macrophages can change their habits and migrate to lymph nodes [121].

Intestinal macrophages also regulate other immune cells. CX3CR1^high^ macrophages capture soluble food antigens and transfer them to CD103^+^ dendritic cells, promoting antigen presentation and food tolerance [141,142]. Lamina propria macrophages produce IL-10, which promotes the differentiation of Forkhead Box P3 (Foxp3)^+^ Tregs [103,118,143], and also produce IL-1β, which acts on Th17 cells [104]. Intestinal macrophages also produce IL-1α and IL-1β in response to commensal microbiota, affecting Group 3 innate lymphoid cells (ILC3), and GM-CSF, which acts on macrophages and dendritic cells to maintain Treg homeostasis [107]. CX3CR1^+^ mononuclear phagocytes prime T cells and promote Th17 cell differentiation [144]. Taken together, these observations show that intestinal macrophages and their secreted cytokines regulate T cell responses in the gut.

#### 2.1.4. Macrophages in the Pathogenesis of IBD

In IBD, the local release of PAMPs and DAMPs at the site of injury triggers intestinal inflammation. During this process, large numbers of Ly6C^high^ inflammatory monocytes are recruited to the intestinal tissue in a process dependent on C-C motif chemokine ligand 2 (CCR2) [89,90,145,146], which is also known as monocyte chemoattractant protein (MCP)-1. Lack of CCR2 in mice abrogates the recruitment of TLR2^+^ CCR2^+^ Gr-1^+^, TNF-α-producing macrophages to the inflamed intestine [147], and reduces symptoms of DSS-induced colitis [147,148] (Figure 2).

Monocyte migration to the lamina propria is also controlled by IL-8 and transforming growth factor (TGF)-β, constitutively generated by mucosal epithelial cells [149].

Mouse models of IBD reveal significantly elevated numbers of macrophages in the colon, characterized by an increase in the proportion of Ly6C^+^ macrophages relative to mature Ly6C^−^ macrophages. This Ly6C^+^ macrophage recruitment is dependent on the expression of CCR2 [90,147]. Interestingly, a pronounced elevation in colonic macrophage numbers, and an increased proportion of Ly6C^+^MHCII^+^ monocyte-derived macrophages are key features of spontaneous colitis in IL-10R-deficient mice on the C57BL/6 background [150].

IBD patients also have an increased number of pro-inflammatory macrophages [90,151,152], and pediatric IBD patients show accumulation of activated mucosal macrophages [153]. Usually, these macrophages have augmented expression of pro-inflammatory molecules such as TNF-α, IL-1β, IL-6, and inducible nitric oxide synthase (iNOS) [151,154]. Recruited Ly6C^high^ monocytes in IBD upregulate TLR2 and NOD2, which increases their sensitivity to bacteria and triggers their differentiation to pro-inflammatory effector cells [122]. These inflammatory macrophages produce TNF-α, IL-6, and iNOS [110,155]. Resident CX3CR1^high^ macrophages maintain their anti-inflammatory phenotype even when sharing the intestine with Ly6C^high^ inflammatory macrophages [90,116].

In steady state conditions, CX3CR1^high^ resident macrophages, which are highly phagocytic and MHCII^high^ but resilient to TLR-stimulation and constitutively IL-10 producers, are accompanied by a small population of CX3CR1^int^ cells, mainly resulting in a CX3CR1^high^ resident macrophage population. CX3CR1^int^ cells give rise to CX3CR1^high^ macrophage. In IBD, this CX3CR1^int^ to CX3CR1^high^ macrophage conversion is diminished, leading to the accumulation of TLR-reactive inflammatory CX3CR1^int^ macrophages [90].

CX3CR1 and its ligand CX3CL1 are upregulated in the colon of IBD mice and seem to play an important role in the disease [156], with CX3CR1 and CX3CL1 polymorphisms in patients linked to the clinical manifestations of IBD [157,158]. However, it is unclear if they play a protective or harmful role, since their deficiency protects from [138] or aggravates [156] experimentally induced colitis depending on the study.

Macrophage-expressed IL-10 and its receptor IL-10R support intestinal homeostasis and are implicated in the development of IBD [159,160,161,162,163,164,165]. IL-10–IL-10R-signaling mediates the differentiation and function of intestinal macrophages in mice and IBD patients [166]. The absence of IL-10 in mice provokes a shift from the resident CX3CR1^high^ macrophage phenotype in the colon to a pro-inflammatory phenotype [121]. Interestingly, specific depletion of IL-10 in CX3CR1^high^ intestinal macrophages has no effect on intestinal homeostasis or Treg regulation [121], but the intestinal macrophage-specific lack of IL-10R alters intestinal homeostasis and provokes severe gut inflammation [121,167]. The absence of Il-10ra in intestinal macrophages promotes the production of IL-23, which in turn mediates IL-22 secretion by Th17 and ILC3 cells. IL-22 activates IECs to express an antimicrobial peptide that induces neutrophil recruitment, promoting IBD [167]. Moreover, colitis symptoms are ameliorated by manipulation of the microbiota to increase IL-10 producing macrophage numbers [109].

Lamina propria macrophages produce the chemokine CCL8, recruiting circulating Ly6C^high^ monocytes in IBD [122,168].

Intestinal macrophages are also important for epithelial tissue repair. These macrophages produce molecules that control epithelial regeneration [169]. As mentioned, intestinal macrophages in the pericryptal stem cell niche stimulate neighboring colonic epithelial progenitors and promote epithelial recuperation after injury [170]. Epithelial recovery is also stimulated by intestinal macrophage-derived IL-10, through the stimulation of the CREB/WISP-1 pathway in epithelial cells [171,172] or by macrophage production of hepatic growth factor (HGF). Curiously, CD-patient-derived macrophages show diminished HGF secretion, possibly affecting epithelial restoration in these patients [173]. Classically, two main polarized macrophage phenotypes have been proposed, pro-inflammatory M1 and anti-inflammatory M2 [174]. Tissue repairing M2-like macrophages promote stimulation of wingless-related integration site (WNT) signaling in response to differentiation driven by signal transducer and activator of transcription (STAT)-6 [175] through a mechanism dependent on the alarmin IL-33 [176]. STAT6-dependent M2-like macrophage differentiation promotes the stimulation of WNT signaling to promote tissue repair [175]. The alarmin IL-33 also favors protective M2-like macrophages polarization and subsequent mucosal repair [176,177]. Another study showed that altered α4β7-mediated intestinal-homing of non-classical monocytes might reduce the number of wound-healing macrophages, leading to impaired intestinal wound healing [178].

Upon tissue injury, macrophages are activated to combat microbiota invasion through phagocytosis and to facilitate repair of the damaged tissue. However, when macrophages are improperly activated, they directly cause the onset and development of fibrosis [179,180]. Fibrosis is a disproportionate accumulation of extracellular matrix (ECM) components such as collagen [181]. Excessive fibrosis produces a non-optimal tissue architecture, and intestinal fibrosis is a common problematic characteristic of IBD [182,183]. Advanced intestinal fibrosis frequently results in intestinal strictures [106]. Macrophages promote myofibroblast-mediated fibrosis by producing TGF-β1, connective tissue growth factor (CTGF), and fibroblast activation protein (FAP). They also undergo macrophage-to-myofibroblast transition (MMT), favoring myofibroblast accumulation and excess ECM production [106]. In contrast, intestinal macrophages can restrict intestinal fibrosis by promoting myofibroblast senescence, degrading the ECM, and clearing profibrotic components [184]. Macrophages are not the only source of gut fibrosis in IBD; IL-34, which is overproduced in IBD and mediates macrophage maturation [125], also activates collagen synthesis by gut fibroblasts [185].

In summary, macrophages play an important role in homeostasis and in the development of IBD [121,150,166,167], both in mouse models and in patients, by phagocytosing cellular debris, producing multiple cytokines, and regulating tissue repair [105].

#### 2.1.5. Innate Lymphoid Cells in IBD

Another important component of the innate immune system in the intestine is the population of innate lymphoid cells (ILCs). ILCs are important mediators of antimicrobial defense and contribute to organ development, tissue protection and regeneration, and mucosal homeostasis [186,187]. These cells belong to the innate immune system, but are derived from the same common lymphoid progenitor population as lymphocytes [188,189]. ILCs act early in the immune response by replying quickly to cytokines and other signals produced by other cells [188,190,191,192,193,194]. Recent discoveries have highlighted the essential role of ILCs in intestinal mucosal homeostasis and IBD [195,196,197,198,199,200]. However, they will be not discussed in this review since we and others have recently reviewed their role in IBD [194,201,202,203,204].

#### 2.1.6. Dendritic Cells in Homeostasis

DCs link the innate and adaptive immune systems by presenting antigens to and activating T cells. Several DC subtypes are derived from a specific common dendritic progenitor (CDP). CDPs generate plasmacytoid DCs (pDCs) in bone marrow, as well as pre-DCs that circulate in the blood and give rise to conventional or classical DCs (cDCs) in lymphoid and nonlymphoid organs [205,206,207,208,209]. The production of pDCs versus cDCs is determined by the growth factor fms-like tyrosine kinase 3 ligand (Flt3L) [210] (Figure 2).

Monocytes originate in the bone marrow and circulate in blood. DCs can also originate from circulating monocytes after monocyte migration to inflamed tissues, where they differentiate to macrophages or monocyte-derived DCs through the action of M-CSF or GM-CSF, respectively. Monocyte-derived DCs belong to the mononuclear phagocyte system (MPS), and are better APCs than monocytes [211,212]. Through their function as professional APCs and their capacity to release cytokines, DCs play important roles in initiating immune responses to invading pathogens; cDCs and monocyte-derived DCs are powerful APCs, whereas pDCs specialize in secreting type I IFN [213]. Moreover, cDC subpopulations included chemokine (C motif) receptor 1 (XCR1)^+^ cDC1s and signal-regulatory protein alpha (SIPRα)^+^ cDC2s [205,206,207,208,209]. cDC2s can be subclassified as CD103^+^ or CD103^−^. These DC subsets possess distinctive functional, phenotypical, and transcriptional features. While cDC1 are excellent APCs to cytotoxic T cells, cDC2s are more similar to pDCs in their capacity to polarize CD4^+^ T cell responses and to promote anti-viral responses via type I IFN [12].

Under physiological conditions, immature DCs patrol peripheral tissues, where they encounter and take up antigens [214]. Upon activation, maturing DCs increase their expression of MHCII and the costimulatory molecules CD80, CD86, and CD83 [209,213] and migrate along a chemokine gradient to draining lymph nodes, where they enter paracortical T cell zones to activate and prime antigen-specific naïve T cells and secrete cytokines [215,216,217,218]. In this way, DCs link innate and adaptive immunity by presenting antigens to and activating T cells.

#### 2.1.7. Dendritic Cells in IBD

DCs accumulate in specific gut locations such as Peyer’s patches, isolated lymphoid follicles, and gut-associated lymphoid tissues. Like macrophages, DCs are constantly replenished from bone marrow-derived progenitors [88] (Figure 2).

Like some macrophages, DCs take up soluble food antigens directly from the intestinal lumen [136], but also take up food antigens from epithelial M-cells in the follicle-associated epithelium of Peyer’s patches [219]. As mentioned, DCs also receive antigens from CX3CR1^high^ lamina propria macrophages through gap junctions [141,142]. In steady state, DCs play a tolerogenic role upon recognizing commensal bacterial components. XCR1^+^ cDC1s are important for intestinal homeostasis and in particular the expression of XCR1; mice lacking XCR1 in cDC1 lack intraepithelial and lamina propria T cell populations, and are more vulnerable to chemically-induced colitis [220].

CD103^+^ cDC2s seem to be important for initiating oral tolerance, in part through their capacity to generate retinoic acid (RA) required for the development of Foxp3^+^ Treg cells [221,222]. Furthermore, mammalian target of rapamycin (mTOR) protein kinase intervenes in the regulation of intestinal homeostasis by enhancing IL-10 production in cDC2s. Indeed, loss of mTOR signaling in DCs blocks IL-10 generation by cDC2s and increases sensitivity to DSS-induced colitis [223].

Colonic DCs display an abnormal immature phenotype in IBD that includes the expression of homing markers [224]. Intestinal DCs from UC patients have diminished expression of cutaneous lymphocyte antigen (CLA) and CCR4, while showing enhanced expression of CCR9 and β7 integrin [225,226].

DCs from CD-patient mucosa express more CD40 and release more IL-6 and IL-12 than DCs from healthy individuals [224]. In IBD, mucosal DCs show increased expression of TLR2 and TLR4 [225]. CD103^+^ CD11b^+^ cDCs are significantly reduced in abundance in the inflamed and uninflamed intestinal tissue of CD patients [227]. pDCs are also found in the inflamed gut, although their specific role is still undetermined [228].

The activation of intestinal CD103^+^ DCs in IBD patients results in the upregulation of PRRs. Thus, local variations in the gut microbiota may change the balance and regulation signals received by mucosal DCs. Upon activation, DCs are able to release inflammatory cytokines [229]. In summary, DCs play an essential role in IBD pathogenesis.

IBD pathogenesis can be augmented by inappropriate macrophage and DC responses to the microbiota [230]. These responses involve inadequate protection and strengthen pathogenicity.

Intestinal DCs promote tolerance to luminal antigens under physiological conditions, but can develop into an inflammatory response after inflammation or direct stimulation by TLR ligands. In these circumstances, intestinal DCs can release inflammatory cytokines such as IL-12, IL-6, and IL-18 and mediate Th1 responses when triggered. Supporting this, the circulation of IBD patients with active disease contains pDCs that migrate to secondary lymphoid organs, where they produce Th1 cytokines (IL-6, IL-8, and TNF-α), thereby perpetuating disease [230]. In addition, inflamed, and uninflamed intestine of CD patients has a reduced abundance of CD11c^+^ DCs, conferring an increased capacity to produce Th1/Th2/Th17 responses [231].

The role of cDC2s in IBD is less clear, with some studies pointing to an implication in T cell-mediated colitis [232,233], while others show no effect of lamina propria CD103^+^ cDC2s [233,234]. However, conditional absence of interferon regulatory factor (IRF)4 in mice results in abnormal development of colon lamina propria cDC2s and late initiation of T cell-dependent colitis [232], indicating a role of IRF4-expressing cDC2s in the initial priming of colitogenic T cells. Remarkably, cDC1s may play a protective role in the development of IBD, since lack of these cells increases predisposition to DSS-induced colitis [220,233].

## 3. Discussion

Comparison of first-degree relatives of IBD patients with the general population reveals a heritable risk of CD and UC [235,236]. Technological advances in genetic testing and DNA sequencing have allowed the development of genome-wide association studies (GWAS), which have identified more than 240 risk variants associated with IBD. These variants are found in genes related to bacteria recognition (e.g., NOD2), autophagy (e.g., ATG16L1 and IRGM), regulation of epithelial barrier (e.g., ECM1), and innate and adaptive immunity (e.g., IL-23R, IL-10, ITGAL, and ICAM1 variants) [237,238]. From these data, it has been possible to uncover fundamental molecular features underlying the disease and to identify genes and signaling pathways that represent potential therapeutic targets or biomarkers. However, only a small percentage of the disease variance in CD and UC can be linked to recognized IBD risk loci [239]. Most GWAS analyses in the field have examined whole intestinal tissue. One consequence of this is that that the data obtained reflect the most highly expressed mRNA transcripts in the more abundant cell populations, thus potentially missing less abundantly expressed genes. Moreover, the obtained data cannot be unambiguously associated with a specific cell population.

To resolve this limitation, new techniques have allowed the study of single-cell-specific transcriptional profiles. For example, single-cell RNA sequencing (scRNA-seq) and high-dimensional protein analyses, such as mass cytometry and multichannel spectral cytometry, have defined IBD-linked profiles and detected cell subpopulations that are elevated or diminished in IBD, particularly populations of fibroblasts [10], epithelial cells [9], and immune cells [240,241,242,243,244,245,246,247,248].

A complementary approach to GWAS is through transcriptome-wide association studies (TWAS), which associate gene expression with genetic susceptibility to disease, providing functional insight into risk loci [249]. TWAS findings have provided understanding of tissue-specific molecular events underlying genetic susceptibility to IBD. Associated genes are potential targets for new treatments and could be prioritized in functional studies.

Neutrophils play a dual role in intestinal homeostasis and inflammation, playing an essential role in gut defense but also, upon excessive recruitment, being an important mediator of tissue damage in the inflamed mucosa. Several studies demonstrate the effect of neutrophils on other components of the intestinal mucosa in IBD, such as other immune cells and epithelial cells and other non-immune cells. However, less is known about the reciprocal effect on neutrophils of these other cells, particularly intra-epithelial cell components as stroma and epithelial cells, although some studies point to the importance of neutrophil–epithelial cell communication for essential neutrophil functions such as recruitment, transepithelial migration, cell death, and clearance (Reviewed in [63]).

Pharmacological inhibition of NET formation ameliorates IBD [39]. Potentiation of the protective functions of neutrophils, in combination with a reduction in the adverse effects of NET formation, offers an interesting approach to the treatment of intestinal diseases; however, this regulation must be achieved without compromising neutrophil immune-defense functions. Targeting NETs is a potentially interesting approach to achieving complete mucosal healing in IBD. Another area of interest for therapeutic intervention is the development of markers of NET formation in vivo. Supporting this, calprotectin (s100A8/9), the most commonly used fecal biomarker in IBD, constitutes up to 60% of neutrophil cytosolic protein content [250,251]. Calprotectin is also found in NETs [36]. Other neutrophil-associated IBD biomarkers of potential therapeutic interest include lactoferrin, CXCR1, CXCR2, MMP-9, NGAL, elafin, HNE, pANCAs, MPO, CD16, CD177, CD64, HNPs, SLPI, and PTX3 [252,253,254].

In addition to the biomarker calprotectin, some other biomarkers have been related to inflammatory bowel disease. The serum levels of the peptide adropin, which acts as an energy regulator through lipid and glucose metabolism [255] and is considered an inflammatory biomarker [256], are reduced in patients with inflammatory bowel diseases, showing a negative correlation with fecal calprotectin [257]. Moreover, serum catestatin levels are increased in patients with inflammatory bowel disease when compared to control subjects [258,259,260]. Catestatin is a peptide proteolytically cleaved from chromogranin A, that primarily acts as an inhibitor of catecholamine secretion, and as stimulator of histamine release [261]. Enterochromaffin cells (EC) in the intestinal epithelium are a major source of chromogranin A [262]. Chromogranin A and catestatin regulate gut permeability via the antagonistic actions of its proteolytic peptides [259]. Catestatin regulates epithelial cell dynamics [263], and alters gut microbiota composition in mice [264]. Human catestatin also regulates intestinal inflammation via the macrophage population and through a STAT-3 dependent pathway in a murine model of colitis [260].

The mucosal immune system is the most extensive part of the immune system. Contrasting the situation in the systemic immune system, intestinal immune cells are involved in a highly balanced immune response aimed at controlling pathogen invasion, while stopping excessive immune responses against innocuous food antigens and commensal microbes that could risk unintentional tissue injury.

Some intestinal cell populations can adjust their functions to the needs of the intestinal microenvironment under steady state, and even modify their phenotype and behavior to adapt to inflammatory conditions. This adaptation can be harmful in IBD, but is also a potential therapeutic target for the treatment of the disease.

This is the case of intestinal macrophages, which restrain their robust pro-inflammatory potential through a natural resistance to producing inflammatory mediators in response to pattern-recognition molecules, while also retaining several of their homeostatic abilities, including scavenging and phagocytosing bacteria, preserving Tregs and maintaining tolerance, and promoting epithelial cell renewal [265]. In the intestinal microenvironment, macrophages adapt their functions to the context. For example, CX3CR1^high^ macrophages can distinguish harmful from commensal bacteria via TLR and NLR recognition. In the intestinal microenvironment, CX3CR1^high^ macrophages are excellent phagocytes, but produce low levels of pro-inflammatory cytokines and maintain tolerance through the production of anti-inflammatory cytokines such as IL-10 [111]. CX3CR1^high^ intestinal macrophages sense and take up bacterial antigen from the intestinal lumen via their transepithelial dendrites [135,136,137,138,139,140]. In homeostasis, the intestinal microbiota inhibit the migration of antigen-loaded CX3CR1^high^ intestinal macrophages to mesenteric lymph nodes, thereby also inhibiting antigen presentation to T cells, and effectively sustaining tolerance towards commensal bacteria.

If the intestinal microbiota is impaired or exposed to inflammatory conditions, CX3CR1^high^ macrophages can differentiate to pro-inflammatory effector cells and acquire the capacity to migrate to lymph nodes and present antigens to lymphocytes, which are critically involved in the development of IBD [121]. However, it is not known whether resident macrophages modify their phenotype during inflammation according to the microenvironment [122,266], or if there are distinct macrophage populations that perform distinct functions [90]. The continuous replenishment of intestinal macrophages from monocytes might facilitate plasticity and adaptation of macrophage populations in response to signals from the local intestinal microenvironment, and this might modify macrophage properties such as migration, cytokine-release profile, antigen presentation, and T and B cell activation, and tissue healing. The use of scRNA-seq helps to define the contribution of several cell types to IBD, including macrophages and other innate immune cells. scRNA-seq studies provide precise knowledge of the spatial immune specialization and dysregulated immune response during IBD at single-cell resolution. For example, this technique has revealed that macrophages and CD8^+^ T cells in the lamina propria of the human colon during ulcerative colitis have an effector phenotype and are activated, while their lipid metabolism is suppressed compared with these cells in the epithelial layer [248]. Given the complexity of the mucosal environment and the peculiarity of its immune populations, tailored therapeutic strategies will require better knowledge of the networks regulating this delicate intestinal balance.

Biological treatments have provided an effective therapeutic advance for many IBD patients; nevertheless, one-third of patients do not respond to therapy (known as primary non-responders). In addition, a subset of patients who initially respond to anti-TNF drugs discontinue therapy because they lose their response (secondary non-responders) or develop intolerance [267,268]. Although these limitations can be explained by low drug levels in the target tissue or immune responses to the treatment, the complexity of IBD pathophysiology is likely to contribute to these treatment failures. Therefore, it is essential to gain a better understanding of the cellular and molecular basis of IBD in order to achieve complete mucosal healing and reversal of inflammation.

## Data Availability

No new data were created in this study. All the data reported in this review were found in original articles cited in the text.

## Abbreviations

APC	Antigen presenting cell
Card9	Caspase recruitment domain 9
CCL	Chemokine (C-C motif) ligand
CCR	C-C motif chemokine receptor
CD	Crohn’s disease
CitH3	Citrullination histone H3
CLA	Cutaneous lymphocyte antigen
CPD	Common dendritic progenitor
CXCL	Chemokine (C-X-C motif) ligand
CX3CR	Chemokine (C-X3-C motif) receptor
DAMPs	Damage-associated molecular patterns
DCs	Dendritic cells
DSS	Dextran sodium sulfate
ECM	Extracellular matrix
Flt3L	Fms-like tyrosine kinase 3 ligand
Foxp3	Forkhead Box P3
G-CSF	Granulocyte colony-stimulating factor
GM-CSF	Granulocyte-macrophage colony-stimulating factor
GWAS	Genome-wide association studies
IBD	Inflammatory bowel disease
IFN	Interferon
Ig	Immunoglobulin
IIC	Innate immune cells
iNOS	Inducible nitric oxide synthetase
ILCs	Innate lymphoid cells
IL	Interleukin
IRF	Interferon regulatory factor
MCP	Monocyte chemoattractant protein
MHCII	Major histocompatibility complex molecules class II
MIP	Macrophage inflammatory proteins
MMPs	Matrix metalloproteinases
MMT	Myofibroblast transition
MPO	Myeloperoxidase
mTOR	Mammalian target of rapamycin
NK	Natural killer
NETs	Neutrophil extracellular traps
NOD2	Nucleotide binding oligomerization domain containing 2
NLR	Nod-like receptors
PAD4	Protein arginine deiminase 4
PAMPs	Pathogen-associated molecular patterns
pDC	Plasmacytoid DCs
RA	Retinoic acid
PRRs	Pattern recognition receptors
RORγt	RAR-related orphan receptor gamma t
SCFAs	Short-chain fatty acids
scRNA-seq	Single cell RNA sequencing
SIPRα	Signal-regulatory protein alpha
STAT	Signal transducer and activator of transcription
Th	T-helper
TGF	Transforming growth factor
TL1A	TNF-like ligand 1 A
TLRs	Toll-like receptors
TNBS	2,4,6-trinitrobenzene sulfonic acid
TNF	Tumor necrosis factor
Treg	T regulatory
Tr1	T regulatory type 1
TWAS	Transcriptome-wide association studies
UC	Ulcerative colitis
WNT	Wingless-related integration site
XCR1	Chemokine (C motif) receptor 1
